# Accuracy of FAST in detecting intraabdominal bleeding in major trauma with pelvic and/or acetabular fractures: a retrospective cohort study

**DOI:** 10.1007/s00590-023-03813-6

**Published:** 2024-01-22

**Authors:** Lasse Rehné Jensen, Emma Possfelt-Møller, Allan Evald Nielsen, Upender Martin Singh, Lars Bo Svendsen, Luit Penninga

**Affiliations:** 1 Department of Surgery and Transplantation, Rigshospitalet, Copenhagen University Hospital, Copenhagen, Denmark; 2 Department of Orthopaedic Surgery, Trauma Section, Rigshospitalet, Copenhagen University Hospital, Copenhagen, Denmark; 3 Department of Clinical Medicine, University of Copenhagen, Copenhagen, Denmark

**Keywords:** Pelvic Fractures, Bleeding, FAST, Laparotomy, Transfusions

## Abstract

**Purpose:**

The Focused Assessment with Sonography for Trauma (FAST) is a tool to rapidly detect intraabdominal and intrapericardial fluid with point-of-care ultrasound. Previous studies have questioned the role of FAST in patients with pelvic fractures. The aim of the present study was to assess the accuracy of FAST to detect clinically significant intraabdominal hemorrhage in patients with pelvic fractures.

**Methods:**

We included all consecutive patients with pelvic and/or acetabular fractures treated our Level 1 trauma center from 2009–2020. We registered patient and fracture characteristics, FAST investigations and CT descriptions, explorative laparotomy findings, and transfusion needs. We compared FAST to CT and laparotomy findings, and calculated true positive and negative findings, sensitivity, specificity, positive predictive value (PPV) and negative predictive value (NPV).

**Results:**

We included 389 patients. FAST had a sensitivity of 75%, a specificity of 98%, a PPV of 84%, and a NPV of 96% for clinically significant intraabdominal bleeding. Patients with retroperitoneal hematomas were at increased risk for laparotomy both because of True-negative FAST and False-positive FAST.

**Conclusion:**

FAST is accurate to identify clinically significant intraabdominal blood in patients with severe pelvic fractures and should be a standard asset in these patients. Retroperitoneal hematomas challenge the FAST interpretation and thus the decision making when applying FAST in patients with pelvic fractures.

**Supplementary Information:**

The online version contains supplementary material available at 10.1007/s00590-023-03813-6.

## Background

The Focused Assessment with Sonography for Trauma (FAST) is a diagnostic tool used in the primary survey of the Advanced Trauma Life Support (ATLS©) algorithm. FAST is applied for rapid detection of bleeding from solid organ lesions [1], and to decide for further evaluation or emergency surgery. Overall, FAST can be performed with high sensitivity, specificity and accuracy to detect free fluid intraabdominally in blunt abdominal trauma [2, 3].

Traumatic pelvic and acetabular fractures account for 3–8% of all fractures. These injuries are associated with high morbidity and mortality. Adverse outcomes are determined by fracture type, severity, and the hemodynamics of the patient [4–6]. Associated injuries and physiological derangement in the multi-traumatized patient increase the risk of worse outcomes [7, 8]. Patients with pelvic fractures can suffer from uncontrolled bleeding from bony surfaces, pelvic venous plexuses, or pelvic arteries located mainly in the retroperitoneum [9, 10]

The accuracy of FAST in patients with traumatic pelvic fractures has been a topic of debate. Some studies have found FAST to be a less reliable tool in patients with pelvic fractures as FAST had a lower specificity and sensitivity to detect intraabdominal bleeding than reported in blunt trauma patients [11, 12]. Though, different definitions and criteria have been applied and may explain the differences between studies [13–15].

We hypothesized that point-of-care (POC) FAST is useful in detecting significant intraabdominal hemorrhage in patients with pelvic fractures. The aim of the present study was to assess the accuracy of FAST to detect and reject clinically significant intraabdominal hemorrhage in patients with severe pelvic fractures.

## Methods

### Study design

We registered POC FAST, which is part of the initial trauma assessment in our institution and are performed by surgical specialists or registrars. Subsequently, we registered CT-scan and laparotomy findings: negative, positive (free fluid in pelvic cavity and/or abdomen), inconclusive or other findings (e.g., retroperitoneal hematoma). These findings were compared to evaluate similarities where CT-scan and laparotomy findings were used as golden standard, that is true positive/negative findings, to either FAST-positive or FAST-negative patients. We then divided patient population into four cohorts based on FAST results: (1) true positive (TruePos), (2) true negative (TrueNeg), (3) false positive (FalsePos), and (4) false negative (FalseNeg). Transfusion units, in the form of red blood cells, plasma and thrombocytes, were used to determine the degree of bleeding of the individual patient and thereby an indicator of hemodynamically (in)stability. We defined major trauma as Injury Severity Score (ISS) > 15 in accordance the literature [16].

### Data source and population

We identified patients with pelvic and acetabular fractures at the Level 1 trauma center, Copenhagen, Denmark in the period from January 1st, 2009 to December 31st, 2020. Patients who were treated conservatively were excluded. Pelvic and acetabular surgery in the Capital and Zealand Regions, Denmark with a total of 2.7 million inhabitants, is centralized at Rigshospitalet. Patients were either admitted primarily at the Level I trauma center or secondarily referred from local or regional hospitals. After identification, we reviewed patient records and registered the following variables: age, sex, date of trauma, type of fracture (pelvic, acetabular or combined), concomitant lesions, ISS, body mass index (BMI), FAST investigations and CT descriptions, explorative laparotomy findings, emergency and final operative interventions, and transfusion history within the first 24 h. All data were registered in Research Electronic Data Capture (REDCap).

### Outcome measures

We assessed true positive and negative FAST findings to determine sensitivity, specificity, positive predictive value (PPV) and negative predictive value (NPV) [17]. Calculations were done twice with (1) true negative FAST if no free fluid were detected on FAST and no operative interventions were needed even if (minimal) free fluid were noted on CT, and (2) false negative FAST if any free fluid was detected on abdominopelvic CT or laparotomy described by attending radiologist and surgeon, respectively. This made it possible to distinguish between the accuracy for significantly intraabdominal bleeding needing intervention *vs* any free fluid e.g., smaller volumes of physiological fluid or minimal bleeding.

### Statistical analysis

Statistical analyses were performed using IBM SPSS version 28. We applied Pearson’s chi-square test with Bonferroni Post Hoc test for categorical data and one-way ANOVA for continuous data to detect statistically significant differences. Correlations were determined by linear regression (Pearson’s correlation). *P* < 0.05 was considered significant.

## Results

### Patient characteristics

**Fig. 1:**
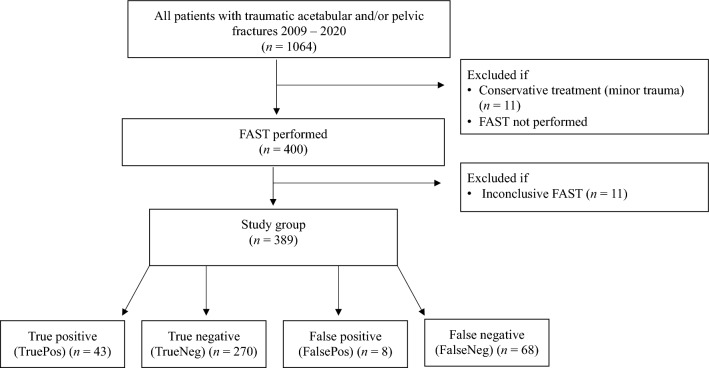
Inclusion of study population

We included 1064 consecutive patients from 2009 to 2020, who were admitted to the Level 1 trauma center, Copenhagen, with a pelvic and/or acetabular fracture. Of these, 400 patients underwent FAST examination. Eleven patients had inconclusive FAST and were excluded for analysis leaving 389 patients for inclusion (Fig. 1). Of these, 203 (52%) had pelvic fractures, 142 (37%) had combined pelvic and acetabular fractures, and 44 (11%) suffered from acetabular fractures. For three patients, we excluded transfusion data as bleeding occurred during emergency orthopedic surgery.

All groups had a mean ISS > 15. We found no significant differences between groups concerning sex, age, fracture type, or BMI. No differences for colon, small intestine or urethra lesions were found between groups. Due to small numbers, no significant differences for specific lesions were detected for FalsePos compared to other groups. A detailed overview is shown in Table 1.

**Table 1:** Patient characteristics of patients undergoing FAST (*n* = 389). *p* < 0.05^†,§,#^, *p* < 0.001^††,§§^

	True FAST-positive	True FAST-negative	False FAST-positive	False FAST-negative	P, Chi.-Sq
n = 389	43 (11% of total n)	270 (69% of total n)	8 (2% of total n)	68 (17% of total n)	
Male sex	26 (60%)	181 (67%)	4 (50%)	48 (71%)	0.53
Age	42.4 (SD ± 19.4)	44.7 (SD ± 19.1)	40.6 (SD ± 13.3)	44.8 (SD ± 20.9)	0.84
Fracture type					
Acetabular	2 (5%)	34 (14%)	1 (13%)	7 (10%)	0.49
Pelvic	19 (44%)	143 (53%)	2 (25%)	39 (57%)	0.23
Combined	22 (51%)	93 (34%)	5 (63%)	22 (32%)	0.06
ISS	34^††^ (SD ± 14)	25^††,§§^ (SD ± 11)	31 (SD ± 15)	36^§§^ (SD ± 14)	< 0.001^††,§§^
BMI	24 (SD ± 4)	25 (SD ± 5)	28 (SD ± 4)	24 (SD ± 4)	0.44
Transfusion received	39^††,††,††,††^ (91%)	132^††^ (49%)	4^††^ (4%)	37^††^ (54%)	< 0.001^††^
Transfusion units (24 h)	22^††^ (SD ± 28)	6^††,§§^ (SD ± 13)	10 (SD ± 16)	16^§§^ (SD ± 29)	< 0.001^††,§§^
Emergency laparotomy	19^††,†^ (44%)	12^††,§§^ (4%)	2 (25%)	14^†,§§^ (21%)	< 0.001^††,§§^ < 0.05^†^
Concomitant lesions					
Liver	18^††^ (42%)	23^††,§^ (9%)	2 (25%)	17^§^ (25%)	< 0.001^††^_,_0.002^§^
Spleen	18^††^ (42%)	14^††,§§^ (5%)	1 (13%)	19^§§^ (28%)	< 0.001^††,§§^
Kidney	8^††^ (19%)	11^††,§§^ (4%)	0	8^§§^ (12%)	0.001^††,§§^
Colon	4^††^ (9%)	3^††,§§^ (1%)	0	4^§§^ (6%)	< 0.001^††,§§^
Small intestine	2 (5%)	0	0	1 (1%)	-
Bladder	5^†^ (12%)	5^†^ (2%)	1 (13%)	2 (3%)	0.003^†^
Urethra	2 (5%)	9 (3%)	0	3 (4%)	0.90
Retroperitoneal hematoma	20^††^ (47%)	53^††^ (20%)	3 (38%)	18 (26%)	< 0.001^††^
None	0	34^§^ (13%)	2^#^ (25%)	1^§,#^ (1%)	< 0.05^§,#^

### Positive FAST

Fifty-one (13% of total population) patients had a positive FAST. Of these, 43 of 51 (11% of total population) were true positive (TruePos) as confirmed by either CT-scan (*n* = 24) or laparotomy findings (*n* = 19). Eight of 51 (2% of total population) patients had a false positive FAST (FalsePos), and of these two patients underwent laparotomy without detectable free fluid. Both patients with FalsePos FAST had a retroperitoneal hematoma.

### Negative FAST

Three hundred and thirty-eight (87% of total population) had a negative FAST. Of these, 270 of 338 (69% of total population) were true negative (TrueNeg) as confirmed by CT (*n* = 258) or laparotomy (*n* = 12). Of these 12 subjects, none of them demonstrated intraabdominal fluid even though nine (75%) had intraabdominal lesions (Table 2). A total of 68 of 338 (17% of total population) patients had a false negative FAST (FalseNeg), and 14 of these underwent a laparotomy.

**Table 2:** Laparotomy findings and retroperitoneal hematomas in patients undergoing emergency laparotomy. TruePos and FalseNeg had clinically significant intraabdominal hemorrhage

Lesion	True FAST-positive *n* = 19	True FAST-negative *n* = 12	False FAST-positive *n* = 2	False FAST-negative *n* = 14	Total (*n* = 47)
Liver	9 (47%)	3 (25%)	1 (50%)	6 (43%)	19 (40%)
Spleen	10 (53%)	2 (17%)	0	4 (29%)	16 (34%)
Kidney	0	1 (8%)	0	2 (14%)	3 (6%)
Colon	3 (16%)	2 (17%)	0	4 (29%)	9 (19%)
Colon mesentery	4 (21%)	3 (25%)	0	0	7 (15%)
Small intestine	2 (11%)	0	0	1 (7%)	3 (6%)
Small intestine mesentery	6 (32%)	1 (1%)	1 (50%)	4 (29%)	12 (26%)
Bladder	2 (11%)	1 (8%)	0	1 (7%)	4 (9%)
Urethra	0	1 (8%)	0	0	1 (2%)
Pancreas	1 (5%)	0	0	1 (7%)	2 (4%)
Diaphragm	1 (5%)	3 (25%)	0	1 (7%)	5 (11%)
Retroperitoneal hematoma	12 (63%)	9 (75%)	2 (100%)	9 (64%)	32 (68%)
Isolated retroperitoneal hematoma	0	2 (17%)	1 (50%)	1 (7%)	4 (9%)
None	0	3 (25%)	0	0	3 (6%)

### Laparotomy findings and FAST

A total of 47 (10% of total population) patients underwent emergency laparotomy due to hemodynamic instability, of which 19 were TruePos, 14 FalseNeg, 12 TrueNeg, and two FalsePos. The most common findings during laparotomy were retroperitoneal hematoma (68%), and lesions of liver (40%), spleen (34%), small intestine mesentery (26%) and colon. Specifically, laparotomy findings for the 14 FalseNeg patients were liver lesion (*n* = 6), splenic lesion (*n* = 4), and large and small intestine lesions (*n* = 4 and *n* = 1). Worth noting, nine (64%) of the FalseNeg patients and nine (75%) of the TrueNeg patients had retroperitoneal hematoma besides intraperitoneal lesions (Table 2).

### Sensitivity and specificity analysis

We established two-by-two tables for all patients assessed by FAST to calculate sensitivity, specificity, positive predictive value (PPV), negative predictive value (NPV), and accuracy. This was done for both (1) need for operative intervention, as an expression of clinically significant bleeding, as true positive (Supplement Table 1) and (2) any free fluid detected on CT or laparotomy as true positive (Supplement Table 2).

Using the need for operative intervention, as an expression of clinically significant bleeding, as true positive resulted in a sensitivity of 75% (95% CI: 62% – 86%), a specificity of 98% (95% CI: 95% – 99%), a PPV of 84% (95% CI: 73% – 92%), a NPV of 96% (95% CI: 94% – 97%), and an accuracy of 94% (95% CI: 92% – 96%) (Supplement Table 1).

Using any volume of free fluid detected on CT or laparotomy as true positive resulted in a sensitivity of 39% (95% CI: 30% – 48%), a specificity of 97% (95% CI: 94% – 99%), a PPV of 84% (95% CI: 72% – 92%), a NPV of 80% (95% CI: 77% – 82%), and an accuracy of 80% (95% CI: 76% – 84%) (Supplement Table 2).

### *Correlation between POC FAST result and transfusion units (*< *24 h)*

Linear regression was significant (*p* < 0.001), and resulted in the following Pearson’s correlations between FAST and transfusion units: 0.2 (95% CI: 0.2% – 0.3%, *p* < *0.001*) for true FAST-positive, 0.2 (95% CI: 0.1% – 0.3%, *p* = *0.003*) for false FAST-negative, 0.001 (95% CI: 0.1% – 0.1%, *p* = *0.99*) for false FAST-positive, and -0.3 (95% CI: −0.2% – −0.4%, *p* < *0.001*) for true FAST-negative.

### *Correlation between concomitant lesions and transfusion units (*< *24 h)*

Linear regression was significant (ANOVA, *p* < 0.001), and resulted in the following Pearson’s correlations between transfusion units and concomitant lesions: 0.4 (95% CI: 0.30% – 0.47%, *p* < 0.001) for retroperitoneal hematoma, 0.3 (95% CI: 0.2% – 0.4%, *p* < 0.001) for colon, 0.3 (95% CI: 0.2% – 0.4%, *p* < 0.001) for liver, 0.3 (95% CI: 0.2% – 0.4%, *p* < 0.001) for spleen, 0.2 (95% CI: 0.1% – 0.3%, *p* < 0.001) for small intestines, 0.2 (95% CI: 0.1% – 0.3%, *p* < 0.001) for urethra, 0.2 for kidney (95% CI: 0.1% – 0.3%, *p* < 0.001), 0.01 for bladder (95% CI: −0.0% – 0.2%, *p* = 0.16), and -0.2 (95% CI: −0.3% – −0.1%, *p* = 0.003) for none (Table 3).

**Table 3:** Pearson’s correlation between concomitant lesions and transfusion units (< 24 h), *p* < 0.05*, *p* < 0.001**

	Correlation, units = 1.0	*p*-value
Liver	0.3 (95% CI: 0.2% – 0.4%)	< 0.001**
Spleen	0.3 (95% CI: 0.2% – 0.4%)	< 0.001**
Kidney	0.2 (95% CI: 0.1% – 0.3%)	< 0.001**
Colon	0.3 (95% CI: 0.2% – 0.4%)	< 0.001**
Small intestine	0.2 (95% CI: 0.1% – 0.3%)	< 0.001**
Bladder	0.01 (95% CI: −0.0% – 0.2%)	0.16
Urethra	0.2 (95% CI: 0.1% – 0.3%)	< 0.001**
Retroperitoneal hematoma	0.4 (95% CI: 0.3% – 0.5%)	< 0.001**
None	−0.2 (95% CI: −0.3% – −0.1%)	0.003*

TrueNeg had significant lower ISS (24 ± 11 SD, *p* < 0.001) and received fewer transfusions units (6 ± 13 SD, *p* < 0.001) compared to TruePos (ISS 34 ± 14 SD, transfusion 22 ± 28 SD) and FalseNeg (ISS 36 ± 14 SD, transfusion 16 ± 29 SD). TruePos and FalseNeg underwent emergency laparotomy significantly more often, 19 (46%) and 14 (21%), respectively, and TrueNeg significantly less corresponding to 12 (5%). TruePos and FalseNeg had significantly more often liver, spleen and kidney lesions (*p* < 0.05) compared to TrueNeg. Furthermore, TruePos had a significantly larger number of urinary bladder lesions and retroperitoneal hematomas (*p* < 0.05) compared to TrueNeg.

## Discussion

### Primary findings

Proper identification of the amount and location of the bleeding in pelvic trauma is of utmost importance*.* In the present study FAST performed in the trauma center setting was an accurate tool to detect clinically significant intraabdominal bleeding with high sensitivity and very high specificity in patients with pelvic and acetabular fractures. FAST was less accurate to detect any free fluid. Hence, the accuracy of FAST is highly dependent on the definition of a true negative FAST. In our opinion, the primary task of FAST is to identify significant intraabdominal bleeding which causes hemodynamically instability and need for acute operative interventions such as emergency laparotomy rather than detection of small insignificant amounts of free fluid.

TruePos FAST was a highly significant predictor for the need for emergency laparotomy, indicating that FAST can identify patients with need of emergency laparotomy.

FAST was also a highly significant predictor for the need for blood transfusion. A significant larger proportion of TruePos FAST received transfusion units compared to all other groups (*p* < 0.001).

We found statistically significant correlations between specific organ lesions: liver, spleen, kidney, and retroperitoneal hematoma and the number of transfusions units. These injuries were all significantly more frequent for the TruePos compared to TrueNeg. This also supports FAST to be an accurate and reliable tool to identify bleeding from abdominal lesions in patients with pelvic fractures. These correlations were weak and should be interpreted with caution but can maybe utilized in the in the overall assessment of the patient group. The correlation was also significant for urethra and small intestine, but we did not find any group differences in these lesions due to few events.

In our study, fourteen patients with hemodynamically instability and a negative FAST underwent laparotomy and intrabdominal fluid was detected. Nine of these 14 patients with false-negative FAST had both a retroperitoneal hematoma and intraabdominal lesions. Large, space-filling retroperitoneal hematomas complicate detection of free intraabdominal fluid: zone II hematomas for spleen and liver and zone III hematomas for pouch of Douglas. Furthermore, it cannot be ruled out that this blood decompressed into the intraabdominal space after initial FAST examination in some cases. Repeated FAST examination is known to increase the sensitivity to detect intraabdominal bleeding in blunt trauma [18]. Thus, another possible explanation for false FAST-negative could be buildup of intraabdominal fluid (e.g., breakthrough from retroperitoneal space or slowly bleeding organ lesions) in the time from POC FAST to CT-scan and or laparotomy.

In contrast, for the 12 patients with true negative FAST undergoing laparotomy, nine (75%) had retroperitoneal hematoma, which could potentially have contributed to hemodynamically instability and prompted laparotomy which have been described in the literature previously [13, 14]. All in all, this paints a picture of retroperitoneal hematomas challenging the interpretation and thus the decision making when applying FAST in this population.

Despite conflicting perspectives in the literature, FAST is still advocated for the initial assessment of trauma patients with pelvic fractures, as emphasized by leading experts [19]. The discrepancies in results across studies might be explained by differences in patient selection e.g., ‘major’ pelvic fractures, definitions of true positive/negative FAST, including for example definitions of significant amount of fluid on CT, and significantly intraabdominal bleeding leading to inter-study heterogeneity. Importantly, the quality of the scan can be influenced by the examiner, whether it is a trained radiologist or a surgeon. Furthermore, if studies permit serial FAST examinations, it could enhance sensitivity [20]. A recent study reported significantly lower sensitivity and specificity compared to our results. However, it is important to note that the study exclusively enrolled hemodynamically unstable patients, which inevitably reduced the number of true negative FAST scans. This difference in patient selection serves as a distinct starting point for the calculations [21]. This does not represent the population on which we conduced the FAST, nor does it consider the significance of a (true) negative FAST result. Therefore, making comparisons with our study is difficult.

The main task of FAST is to detect intraabdominal bleeding in hemodynamically instable patients with need of emergency intervention [14, 15, 22], by laparotomy, and not by detecting specific abdominal injuries as some studies suggest [23]. A recent meta-analysis corroborates our findings. It defined significant intraabdominal injury as an injury necessitating surgical intervention through abdominal exploration. The study concluded that the FAST accurately identified significant intraabdominal hemorrhage in patients with pelvic fractures [24]. Consequently, we concur with the authors that, in this patient population, the evaluation of the FAST should not focus on its ability to detect any free fluid, as a positive FAST is defined in accordance with the ATLS guidelines. Further, the magnitude of hemoperitoneum predicts the need for surgical hemorrhage control [25], which suggest a role for FAST, as the sensitivity increases with increasing volumes of intraabdominal fluid [26, 27]. Solid-organ injuries can be diagnosed with ultrasound, but this is quite challenging and offers low sensitivity [28]. If patients with negative FAST are hemodynamically stable, a CT should be done to detect possible specific lesions.

The primary strength of our study was inclusion of all consecutive, unselected patients over a 11-year period in our institution. We only excluded very few patients because of insufficient medical records and patients with conservative treated pelvic fractures. The study is based on a detailed clinical data set comprising patient characteristics, concomitant lesions, interventions/procedures, and transfusion data on meticulous review of the medical records and diagnostic radiology of all patients. In addition, we correlated FAST results to transfusion units, and linked transfusion needs (< 24 h) to specific lesions which illuminates important clinical factors related to FAST results.

The study also had certain limitations. First, this was a retrospective study, containing the natural limitations that follow, even though some data from the registry were prospectively registered. Furthermore, FAST examination and interpretation is affected by a learning curve and has a high degree of inter-observer variability which may have implications for the transfer from theory to practice [29, 30].

## Conclusion

FAST is an accurate procedure to detect or reject the presence of clinically significant intraabdominal blood in patients with pelvic fractures. Retroperitoneal hematomas challenge the interpretation and thus the decision making when applying FAST in patients with pelvic fractures.

### Supplementary Information

Below is the link to the electronic supplementary material.

Supplementary file1 (DOCX 13 KB)

Supplementary file2 (DOCX 13 KB)

## Data Availability

The datasets used and/or analyzed during the current study are available from the corresponding author on reasonable request.
