# Supplementary material for: Accuracy of FAST in detecting intraabdominal bleeding in major trauma with pelvic and/or acetabular fractures: a retrospective cohort study
Source: Eur J Orthop Surg Traumatol. 2024 Jan 22;34(3):1479–86. doi: 10.1007/s00590-023-03813-6 (PMC10980602; doi:10.1007/s00590-023-03813-6)
Supplement: Supplementary file 1 — Supplementary file1 (DOCX 13 KB) [file 590_2023_3813_MOESM1_ESM.docx]

**Supplement Table 1** Sensitivity and specificity analysis: Need for operative intervention in negative FAST

| Test |  | n | |  | n | Total | | |
| --- | --- | --- | --- | --- | --- | --- | --- | --- |
| Positive | True positive | 43 | | False positive | 8 | 51 | | |
| Negative | False negative | 14 | | True negative | 324 | 338 |  |  |
| Total | Total | 57 |  | | | 332 | |  |
