# Supplementary material for: Accuracy of FAST in detecting intraabdominal bleeding in major trauma with pelvic and/or acetabular fractures: a retrospective cohort study
Source: Eur J Orthop Surg Traumatol. 2024 Jan 22;34(3):1479–86. doi: 10.1007/s00590-023-03813-6 (PMC10980602; doi:10.1007/s00590-023-03813-6)
Supplement: Supplementary file 2 — Supplementary file2 (DOCX 13 KB) [file 590_2023_3813_MOESM2_ESM.docx]

**Supplement Table 2** Sensitivity and specificity analysis: Any volume free fluid detected on CT or laparotomy

| Test |  | n |  | n | Total |
| --- | --- | --- | --- | --- | --- |
| Positive | True positive | 43 | False positive | 8 | 51 |
| Negative | False negative | 68 | True negative | 270 | 338 |
| Total | Total | 111 |  | 278 |  |
